# Supplementary figures and images for: Loss of systemic anti-viral immunity and LMP1-driven suppressive myeloid tumour niches converge to shape the immunobiology of Epstein-Barr virus-positive diffuse large B-cell lymphoma
Source: Leukemia. 2026 Jun 10;40(8):1676–87. doi: 10.1038/s41375-026-02994-3 (PMC13421338; doi:10.1038/s41375-026-02994-3)

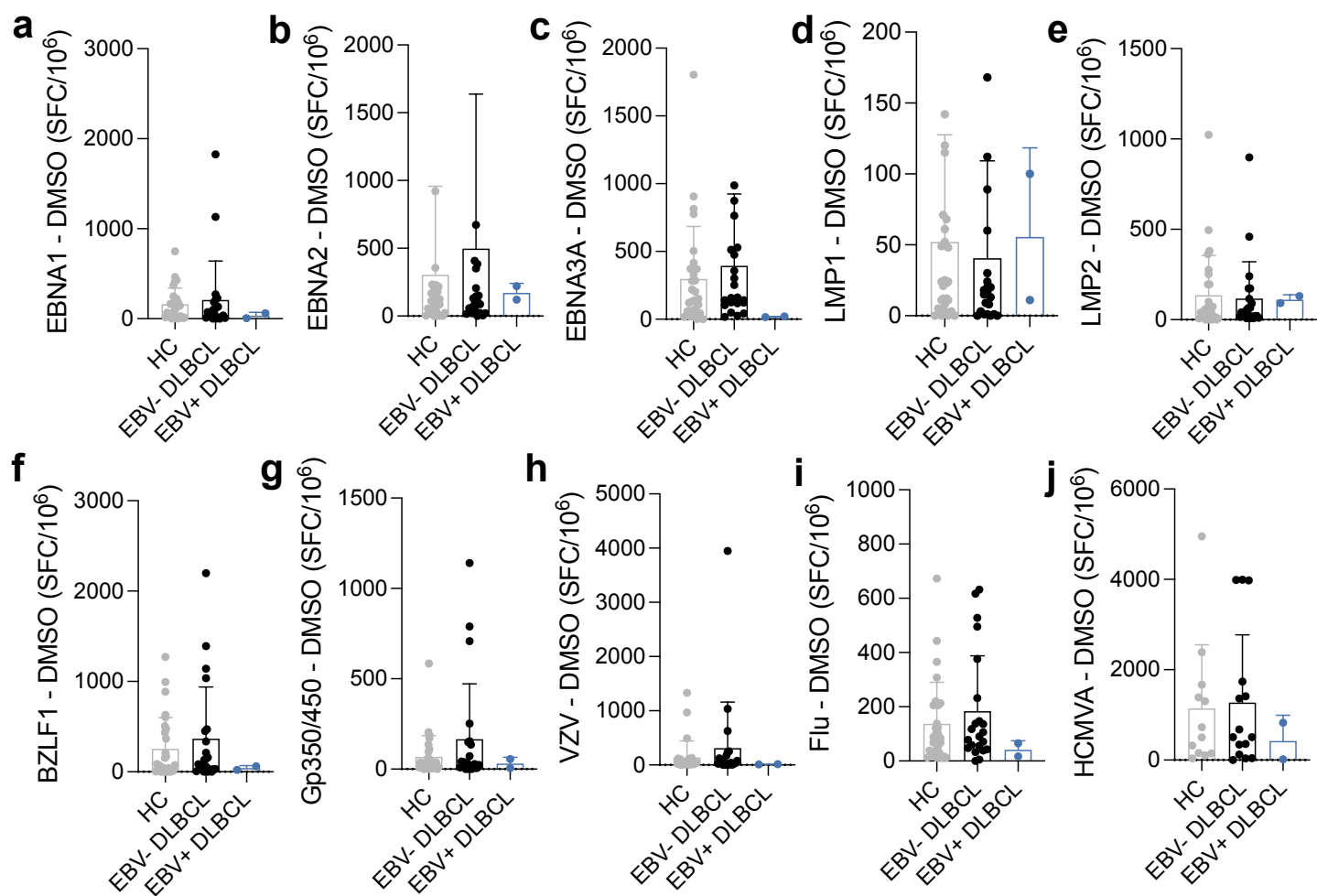

Supplement: Supplementary file 1 — Suppl Figure 1 [file 41375_2026_2994_MOESM1_ESM.pdf]

**a**

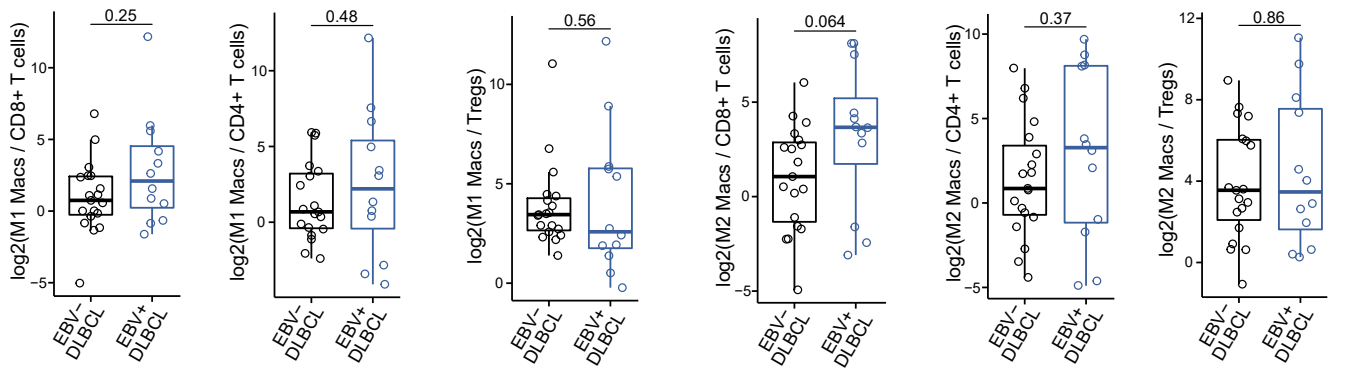

**b.**

### Cell Neighbourhoods (mlF)

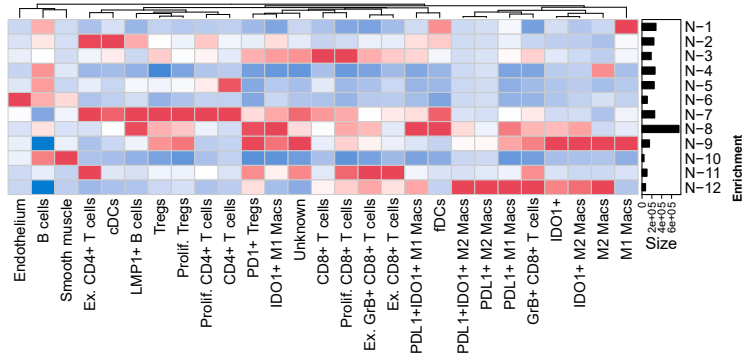

**C.**

Chemokines (RNAseq)

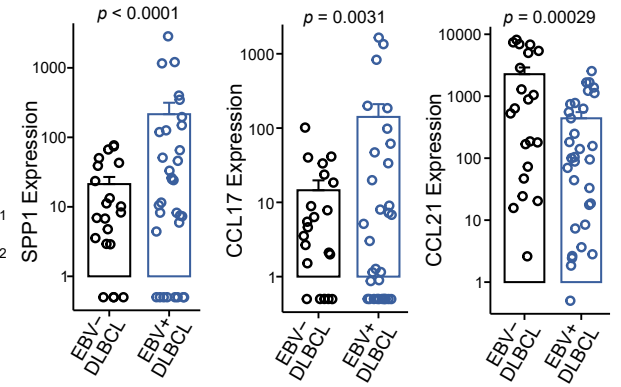

Supplement: Supplementary file 3 — Suppl Figure 3 [file 41375_2026_2994_MOESM3_ESM.pdf]

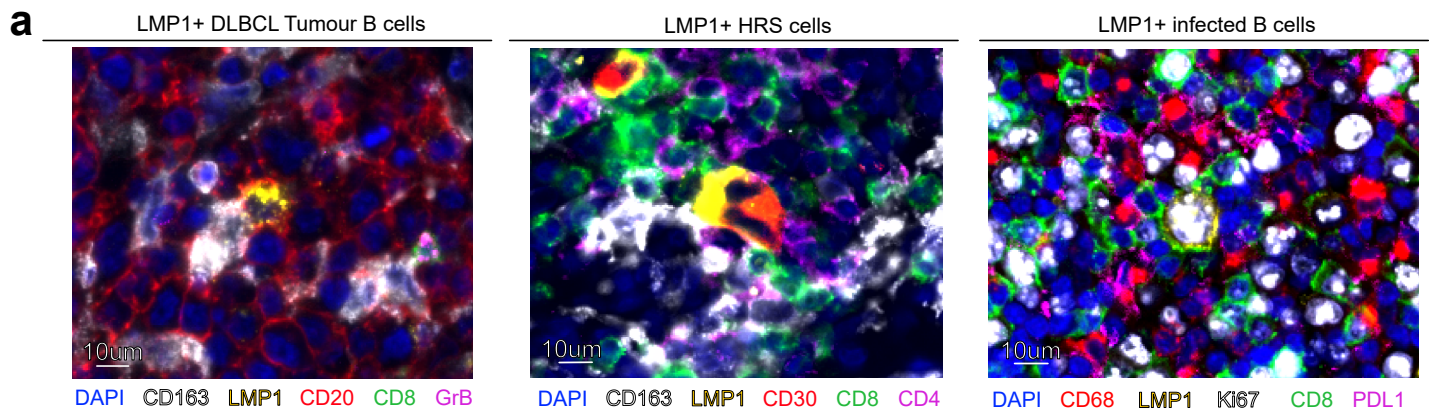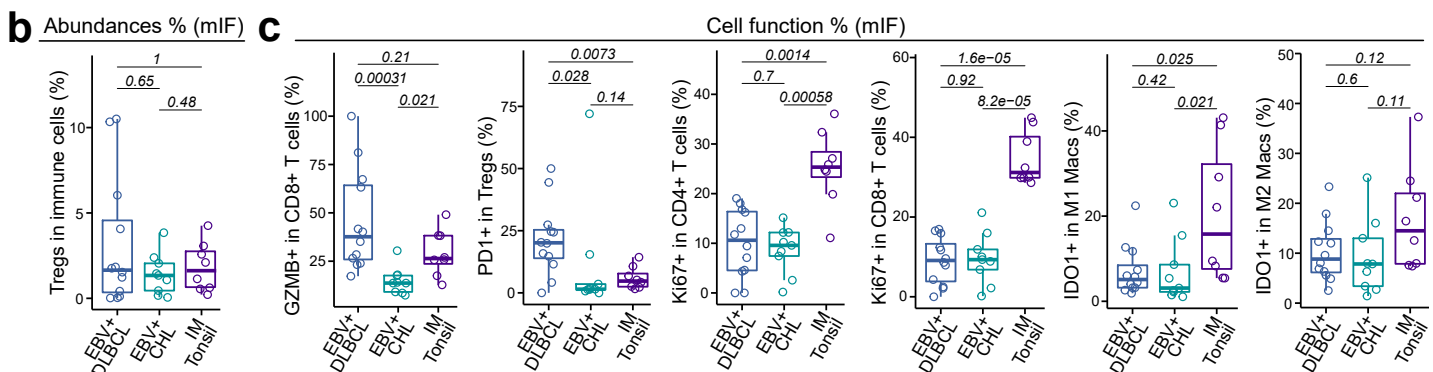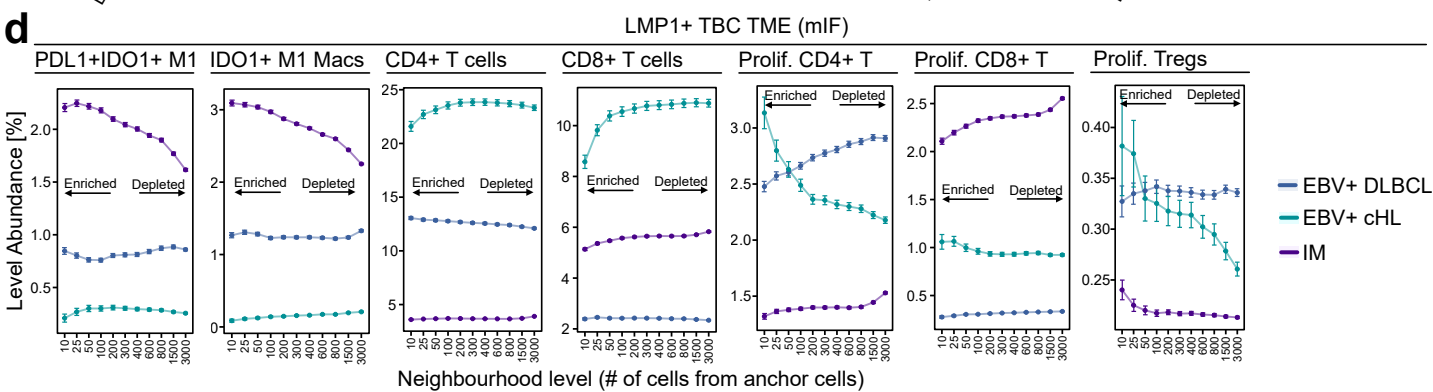

Supplement: Supplementary file 5 — Suppl Figure 5 [file 41375_2026_2994_MOESM5_ESM.pdf]
