# Supplementary material for: Loss of systemic anti-viral immunity and LMP1-driven suppressive myeloid tumour niches converge to shape the immunobiology of Epstein-Barr virus-positive diffuse large B-cell lymphoma
Source: Leukemia. 2026 Jun 10;40(8):1676–87. doi: 10.1038/s41375-026-02994-3 (PMC13421338; doi:10.1038/s41375-026-02994-3)
Supplement: Supplementary file 2 — Suppl Figure 2 [file 41375_2026_2994_MOESM2_ESM.pdf]

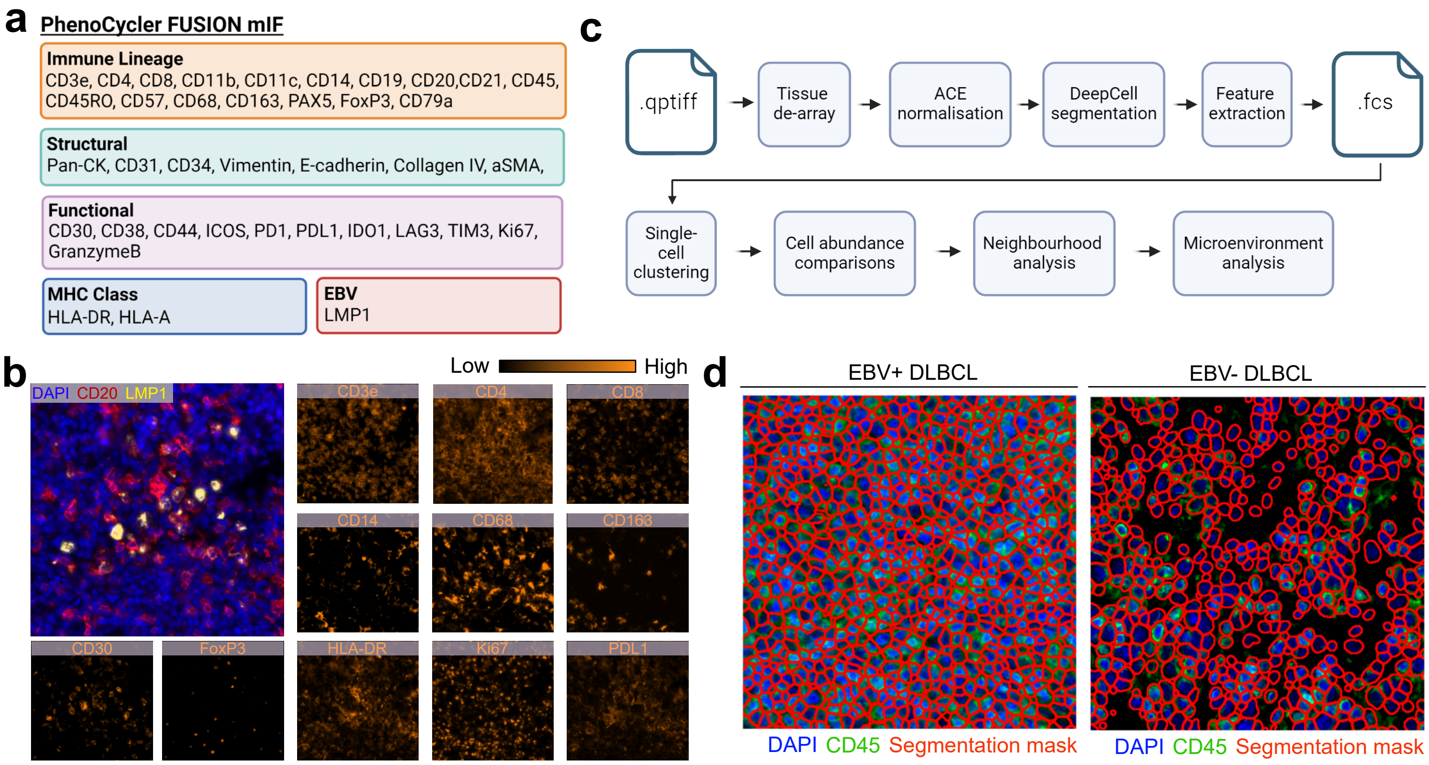
a
PhenoCycler FUSION mIF
Immune Lineage
CD3e, CD4, CD8, CD11b, CD11c, CD14, CD19, CD20,CD21, CD45, CD45RO, CD57, CD68, CD163, PAX5, FoxP3, CD79a
Structural
Pan-CK, CD31, CD34, Vimentin, E-cadherin, Collagen IV, aSMA,
Functional
CD30, CD38, CD44, ICOS, PD1, PDL1, IDO1, LAG3, TIM3, Ki67, GranzymeB
MHC Class
HLA-DR, HLA-A
EBV
LMP1
b
Low
High
DAPI CD20 LMP1
CD3e
CD4
CD8
CD14
CD68
CD163
CD30
FoxP3
HLA-DR
Ki67
PDL1
c
.qptiff
Tissue de-array
ACE normalisation
DeepCell segmentation
Feature extraction
.fcs
Single-cell clustering
Cell abundance comparisons
Neighbourhood analysis
Microenvironment analysis
d
EBV+ DLBCL
EBV- DLBCL
DAPI CD45 Segmentation mask
DAPI CD45 Segmentation mask
