# Supplementary material for: Loss of systemic anti-viral immunity and LMP1-driven suppressive myeloid tumour niches converge to shape the immunobiology of Epstein-Barr virus-positive diffuse large B-cell lymphoma
Source: Leukemia. 2026 Jun 10;40(8):1676–87. doi: 10.1038/s41375-026-02994-3 (PMC13421338; doi:10.1038/s41375-026-02994-3)
Supplement: Supplementary file 6 — Supplementary Figure Legends [file 41375_2026_2994_MOESM6_ESM.docx]

**Extended Data Figure Legends**

**Extended Data Figure 1 | Frequency of T-cells specific for viral antigens in EBV^+^ and EBV^-^DLBCL patients compared with healthy controls (validation cohort)**

**a - j.** IFNγ ELISpot response to latent and lytic EBV antigens as well as control viruses (VZV, Influenza & CMV). Latent and lytic EBV antigen EBNA1, EBNA2, EBNA3A, LMP1, LMP2 and EBV lytic antigen BZLF1 and GP350/450 peptide pools were tested in PBMCs from heathy controls (grey), EBV^-^ DLBCL (black) and EBV^+^ DLBCL (blue), presented as spot-forming cells (SFC) per 10^6^ PBMCs.

**Extended Data Figure 2 | Overview of mIF panel, image pre-processing, and segmentation**

**a.** Phenocycler-FUSION antibody panel

**b.** Example Phenocycler-FUSION staining of CD3e, CD4, CD8, CD14, CD68, CD163, CD30, FoxP3, HLA-DR, Ki67 and PDL1 from a representative field of view of EBV^+^ DLBCL.

**c.** mIF image pre-processing and analysis pipeline used for Phenocycler-FUSION images.

**d.** Examples of single-cell segmentation using DeepCell.

**Extended Data Figure 3 | Additional EBV^+^ TME data**

**a.** Ratios of Macrophage and T-cell phenotypes between EBV^+^ and EBV^-^ DLBCL.

**b.** Cellular neighbourhood cell enrichment heatmap showing the relative abundances of cell types within each neighbourhood.

**c.** Chemokine levels from bulk RNAseq analysis in EBV^+^ vs. EBV^-^ DLBCL.

**Extended Data Figure 4 | Spatial niches of macrophages and LMP1^+^ TBCs in EBV^+^ DLBCL**

**a.** LMP1 transfection and EBV infection of four human DLBCL cell lines (SUDHL4, SUDHL5, HT & U2392).

**b.-c.** EBV gene expression of (e) EBV infected and (f) LMP1 transfected hDLBCL cell lines.

**d.** Commonly upregulated genes in LMP1 transfected and EBV infected DLBCL cell lines.

**e.** TME quantification (cell abundance as a function of distance) of LMP1^+^ TBCs for M1 macrophages, PDL1^+^IDO1^+^ M1 macrophages, PDL1^+^ M2 macrophages, M2 macrophages, exhausted CD4^+^ T-cells, exhausted CD8^+^ T-cells, CD4^+^ T-cells and PD1^+^ T regs.

**f. & g.** TME quantification (cell abundance as a function of distance) of (b) PDL1^+^ M1 macrophage for PDL1^+^IDO1^+^ M1 macrophages, PDL1^+^IDO1^+^ M2 macrophages and PD1^+^ Tregs and (c) PDL1^+^ M2 macrophages for CD4^+^ T-cells, CD8^+^ T-cells and Tregs. Kruskal-wallis test was used at each TME level.

**Extended Data Figure 5 | EBV^+^ disease- mIF images and microenvironment**

**a.** Representative mIF images of different TMEs of EBV^+^ DLBCL TBCs, EBV^+^ cHL HRS cells and EBV-infected B-cells in IM Tonsil.

**b.** Treg abundances as a function of total immune cells in EBV^+^ DLBCL, EBV^+^ cHL & IM Tonsil.

**c.** GranzymeB^+^ CD8^+^ T-cell, PD1^+^ Treg, Ki67^+^ CD4^+^ T-cell, Ki67^+^ CD8^+^ T-cell, IDO1^+^ M1 macrophage and IDO1^+^ M2 macrophage abundances as a function of total immune in EBV^+^ DLBCL, EBV^+^ cHL & IM Tonsil.

**d.** TME quantification (cell abundance as a function of distance) of LMP1^+^ TBCs for PDL1^+^IDO1^+^ M1 macrophages, IDO1^+^ M1 macrophages, CD4^+^ T-cells, CD8^+^ T-cells, proliferative CD4^+^ cells, proliferative CD8^+^ T-cells and proliferative Tregs.
