# Supplementary material for: Loss of systemic anti-viral immunity and LMP1-driven suppressive myeloid tumour niches converge to shape the immunobiology of Epstein-Barr virus-positive diffuse large B-cell lymphoma
Source: Leukemia. 2026 Jun 10;40(8):1676–87. doi: 10.1038/s41375-026-02994-3 (PMC13421338; doi:10.1038/s41375-026-02994-3)
Supplement: Supplementary file 7 — Supplementary Tables [file 41375_2026_2994_MOESM7_ESM.docx]

**Fennell et al Supplementary Tables**

**Table S1: Discovery Blood Cohort**

| **ID** | **Disease** | **Age** | **Sex** |
| --- | --- | --- | --- |
| BM048 | EBV+ DLBCL | 62 | M |
| BM053 | EBV+ DLBCL | 24 | F |
| BM078 | EBV+ DLBCL | 56 | M |
| BM153 | EBV+ DLBCL | 45 | F |
| NHL21 041 | EBV+ DLBCL | 53 | F |
| BM003 | DLBCL | 57 | M |
| BM066 | DLBCL | 84 | M |
| BM110 | DLBCL | 58 | M |
| BM111 | DLBCL | 85 | M |
| BM117 | DLBCL | 69 | M |
| BM119 | DLBCL | 81 | F |
| BM140 | DLBCL | 57 | M |
| BM148 | DLBCL | 45 | M |
| BM150 | DLBCL | 68 | M |
| BM156 | DLBCL | 56 | F |
| BM162 | DLBCL | 72 | M |
| BM202 | DLBCL | 66 | M |
| BM208 | DLBCL | 77 | F |
| BM225 | DLBCL | 78 | F |
| BM232 | DLBCL | 55 | M |
| BM237 | DLBCL | 75 | F |
| BM242 | DLBCL | 56 | F |
| BM251 | DLBCL | 81 | M |
| BM252 | DLBCL | 35 | F |
| BM272 | DLBCL | 62 | M |
| BM278 | DLBCL | 71 | M |
| BM279 | DLBCL | 44 | M |
| BM285 | DLBCL | 60 | M |
| BM289 | DLBCL | 31 | M |
| BM297 | DLBCL | 83 | F |
| BrJa70 | Healthy | 44 | M |
| DaPr63 | Healthy | 51 | F |
| HC005 | Healthy | 22 | M |
| HC013 | Healthy | 29 | F |
| HC018 | Healthy | 32 | F |
| HC025 | Healthy | 30 | F |
| AnFi80 | Healthy | 32 | M |
| GeTh66 | Healthy | 49 | M |
| HC001 | Healthy | 29 | F |
| HC002 | Healthy | 56 | F |
| HC003 | Healthy | 42 | M |
| HC006 | Healthy | 35 | F |
| HC009 | Healthy | 35 | M |
| HC012 (F) | Healthy | 49 | F |
| HC012 (M) | Healthy | 54 | M |
| HC014 | Healthy | 22 | F |
| HC017 | Healthy | 31 | F |
| HC019 | Healthy | 29 | F |
| HC024 | Healthy | 34 | M |
| HC029 | Healthy | 36 | M |
| HC036 | Healthy | 36 | M |

**Table S2 Validation Blood Cohort**

| ID | Disease | Age | Sex | Stage |
| --- | --- | --- | --- | --- |
| IP001 | DLBCL | 77 | M | IES |
| IP002 | DLBCL | 68 | F | primary CNS - no Ann Arbor score |
| IP003 | DLBCL | 68 | F | intra spinal - no Ann Arbor score |
| IP004 | DLBCL | 58 | M | IVA |
| IP005 | DLBCL | 66 | F | 2B |
| IP006 | DLBCL | 46 | M | Stage 4(Liver) |
| IP007 | DLBCL | 63 | M | Stage 4(Pleura) |
| IP008 | DLBCL | 32 | M | IIEA |
| IP009 | DLBCL | 76 | M | Stage 3 |
| IP010 | DLBCL | 76 | M | Stage llB |
| IP011 | DLBCL | 73 | M | Stage IV B |
| IP012 | DLBCL | 78 | F | Stage II |
| IP013 | EBV+ DLBCL | 77 | M | III |
| IP014 | DLBCL | 78 | F | Stage 4 (Pleura) |
| IP015 | DLBCL | 53 | M | Stage 3S |
| IP016 | EBV+ DLBCL | 67 | F | IV |
| IP017 | DLBCL | 66 | F | II |
| IP018 | DLBCL | 69 | M | IAe |
| IP019 | EBV+ DLBCL | 65 | F | Stage 3S |
| IP020 | DLBCL | 79 | F | IVA |
| IP021 | DLBCL | 70 | M | stage 3 |
| IP022 | DLBCL | 70 | F | - |
| IP023 | EBV+ DLBCL | 83 | F | IVB |
| IP024 | DLBCL | 68 | F | IVa |
| IP025 | EBV+ DLBCL | 71 | M | stage 4 |
| IP026 | DLBCL | 59 | F | IV |
| IP027 | DLBCL | 44 | F | Stage IIIB |
| IP028 | DLBCL | 62 | F | IBe |
| IP031 | DLBCL | 43 | M | stage 4 |
| IP032 | DLBCL | 71 | F | 1E |
| IP034 | DLBCL | 76 | M | 1V A |
| IP036 | EBV+ DLBCL | 77 | M | Stage 4 |
| IP038 | DLBCL | 68 | F | Stage 4 |
| IP039 | DLBCL | 82 | M | lA |
| IP040 | DLBCL | 83 | M | stage 4 |
| IP041 | DLBCL | 71 | M | Stage 1AE (Gastric) |
| IP042 | DLBCL | 46 | M | 4S |
| IP043 | EBV+ DLBCL | 67 | M | stage 2 |
| IP044 | DLBCL | 84 | M | 2Be |
| IP045 | DLBCL | 58 | M | 1A |
| IP046 | DLBCL | 68 | M | IVB |
| IP048 | DLBCL | 65 | F | Iae |
| IP049 | DLBCL | 59 | M | 1A |
| IP050 | DLBCL | 70 | F | 111S |
| IP051 | DLBCL | 65 | F | IVA |
| IP052 | DLBCL | 78 | M | IV |
| IP053 | DLBCL | 54 | M | IB |
| IP054 | DLBCL | 19 | M | IE |
| IP055 | DLBCL | 71 | F | IIIE |
| hIP003 | Healthy | 74 | M | - |
| hIP004 | Healthy | 73 | F | - |
| hIP005 | Healthy | 73 | F | - |
| hIP007 | Healthy | 73 | M | - |
| hIP008 | Healthy | 69 | M | - |
| hIP009 | Healthy | 73 | M | - |
| hIP010 | Healthy | 76 | F | - |
| hIP015 | Healthy | 71 | M | - |
| hIP016 | Healthy | 73 | M | - |
| hIP018 | Healthy | 79 | M | - |
| hIP022 | Healthy | 75 | M | - |
| hIP029 | Healthy | 68 | F | - |
| hIP031 | Healthy | 72 | M | - |
| hIP034 | Healthy | 73 | F | - |
| hIP035 | Healthy | 70 | M | - |
| hIP044 | Healthy | 47 | F | - |
| hIP045 | Healthy | 70 | M | - |
| hIP046 | Healthy | 45 | M | - |
| hIP047 | Healthy | 58 | M | - |
| hIP048 | Healthy | 58 | M | - |
| hIP049 | Healthy | 45 | M | - |
| hIP050 | Healthy | 53 | F | - |
| hIP051 | Healthy | 18 | M | - |
| hIP040 | Healthy | 63 | F | - |
| hIP042 | Healthy | 65 | F | - |
| hIP043 | Healthy | 62 | F | - |
| hIP021 | Healthy | 70 | F | - |
| hIP027 | Healthy | 75 | F | - |

**Table S3: ELISPOT Panel**

| **Peptide pool** | **Virus** | **Antigen(s)** | **Protein ID (swiss prot)** | **Supplier** |
| --- | --- | --- | --- | --- |
| **EBNA1** | **Epstein-Barr virus (EBV)** | **Epstein–Barr nuclear antigen 1** | **P03211** | **JPT Peptide Technologies GmbH** |
| **EBNA2** | **Epstein-Barr virus (EBV)** | **Epstein–Barr nuclear antigen 2** | **P12978** | **JPT Peptide Technologies GmbH** |
| **EBNA3A** | **Epstein-Barr virus (EBV)** | **Epstein–Barr nuclear antigen 3A** | **P12977** | **JPT Peptide Technologies GmbH** |
| **LMP1** | **Epstein-Barr virus (EBV)** | **Latent Membrane Protein 1** | **P03230** | **JPT Peptide Technologies GmbH** |
| **LMP2** | **Epstein-Barr virus (EBV)** | **Latent Membrane Protein 2** | **P13285** | **JPT Peptide Technologies GmbH** |
| **BZLF1** | **Epstein-Barr virus (EBV)** | **BamHI Z fragment leftward open reading frame 1** | **P03206** | **JPT Peptide Technologies GmbH** |
| **GP350** | **Epstein-Barr virus (EBV)** | **Epstein-Barr virus glycoprotein 350** | **P03200** | **JPT Peptide Technologies GmbH** |
| **FLU** | **Influenza A** | **MP1 (California (H1N1)), NP (H3N2)** | **C3W5Z8, O91743** | **JPT Peptide Technologies GmbH** |
| **VZV** | **Varicella zoster virus** | **Glycoprotein E (gE)** | **P09259** | **JPT Peptide Technologies GmbH** |
| **HHV-6** | **Human herpesvirus 6** | **U54** | **Q9QJ29** | **JPT Peptide Technologies GmbH** |
| **CMV** | **Cytomegalovirus** | **Immediate Early-1** | **P13202** | **JPT Peptide Technologies GmbH** |

**Table S4: Phenocycler Fusion Panel**

| **Marker** | **Clone** | **Channel** |
| --- | --- | --- |
| CD4 | EPR6855 | AF647 |
| CD68 | KP1 | AF647 |
| CD20 | L26 | AF750 |
| CD11c | 118/A5 | AF647 |
| CD8 | C8/144B | ATTO550 |
| HLA-DR | EPR3692 | AF647 |
| CD3e | EP449E | AF647 |
| CD44 | 156-3C11 | ATTO550 |
| CD45 | D9M8I | AF647 |
| HLA-A | EP1395Y | AF750 |
| CD14 | EPR3653 | AF750 |
| Ki67 | B56 | ATTO550 |
| Pan-cytokeratin | AE1-AE3 | AF750 |
| CD57 | HNK-1 | ATTO550 |
| CD45RO | UCHL1 | ATTO550 |
| CD163 | D6U1J | AF647 |
| CD19 | RM332 | ATTO550 |
| Foxp3 | 259D/C7 | AF647 |
| Granzyme B | D6E9W | ATTO550 |
| CD11b | EP1345Y | AF647 |
| CD21 | EP3093 | ATTO550 |
| PAX5 | RM322 | ATTO550 |
| CD79a | RM297 | ATTO550 |
| CD38 | RM388 | AF750 |
| E-cadherin | 4A2C7 | ATTO550 |
| SMA | 1A4 | AF750 |
| Vimentin | 091D3 | AF750 |
| Collagen IV | EPR209660 | AF647 |
| CD34 | QBEND/10 | AF750 |
| CD31 | EP3095 | AF750 |
| PD-1 | D4W2J | AF647 |
| PDL1 | RM320 | ATTO550 |
| ICOS | D1K2T | ATTO550 |
| TIM3 | D5D5R | AF647 |
| LAG3 | D2G40 | ATTO550 |
| IDO1 | V1NC3IDO | ATTO550 |
| LMP1 | CS1-4 | ATTO550 |
| CD30 | BLR055F | AF647 |

**Table S5: LMP1 RNAseq_UP**

| Gene |
| --- |
| ALDOC |
| BIRC3 |
| RHOG |
| ARSB |
| BCL2A1 |
| BCL2L1 |
| CASP10 |
| CD80 |
| CD40 |
| CD44 |
| CD58 |
| CD70 |
| LYST |
| CCR7 |
| COL1A1 |
| KLF6 |
| CYP1A2 |
| DNMT3A |
| DUSP1 |
| DUSP2 |
| GPR183 |
| EGR2 |
| PTK2B |
| NR6A1 |
| GPD1 |
| MKNK2 |
| RAPGEF1 |
| HLA-DPA1 |
| HLA-DQA1 |
| HLA-DQB1 |
| HLA-DRA |
| HLA-DRB1 |
| HLA-DRB5 |
| HLA-F |
| HLA-L |
| IGSF3 |
| ICAM1 |
| IFNGR2 |
| IL2RA |
| IL2RB |
| IRF5 |
| JAK2 |
| JUNB |
| CD82 |
| KCNN1 |
| KCNN4 |
| LCP1 |
| SMAD7 |
| CIITA |
| GADD45B |
| MYO5B |
| NCF2 |
| NFKB1 |
| NFKB2 |
| NFKBIA |
| NFKBIE |
| NGFR |
| OXTR |
| P2RX7 |
| CFP |
| PFKFB4 |
| PIK3CD |
| MAPK11 |
| RAP2A |
| RARA |
| RDX |
| RGS1 |
| FSCN1 |
| SRC |
| TAP1 |
| TSPAN4 |
| GPR137B |
| TMSB4X |
| TNF |
| TNFAIP3 |
| TRAF1 |
| TYK2 |
| CLIP2 |
| ZFP36 |
| TFEB |
| NCK2 |
| SEMA7A |
| BCAS1 |
| BHLHE40 |
| SCARF1 |
| PLA2G4C |
| SOCS1 |
| TNFRSF14 |
| RIPK2 |
| CD84 |
| ST3GAL5 |
| IER3 |
| UNC119 |
| MTMR4 |
| SYNGR3 |
| GPR55 |
| TRIP10 |
| NR1D1 |
| IER2 |
| PHACTR2 |
| TSC22D2 |
| MVP |
| HRSP12 |
| DENND4A |
| BTN2A2 |
| IFI30 |
| STAG3 |
| CYSLTR1 |
| ARID5A |
| C10orf10 |
| DUSP10 |
| SYNPO |
| HSPA4L |
| FNBP1 |
| KCNH4 |
| DDX58 |
| PPP1R15A |
| ABTB2 |
| ZBTB32 |
| PDLIM3 |
| DEXI |
| WDR91 |
| SNX11 |
| HILPDA |
| GPR132 |
| IER5 |
| SNX9 |
| LINC00158 |
| TMX3 |
| CPVL |
| DDIT4 |
| IL17RD |
| CFAP46 |
| NDE1 |
| FNBP1L |
| ENOX1 |
| KIF26B |
| FLVCR2 |
| DNAH7 |
| SERTAD4 |
| ERO1B |
| DUSP22 |
| TMCC3 |
| ARHGAP31 |
| SLAMF7 |
| SQRDL |
| POPDC2 |
| SAMSN1 |
| FNDC3B |
| ZBTB10 |
| AHNAK |
| CCDC28B |
| TNIP2 |
| TBC1D17 |
| VASH2 |
| FRAS1 |
| CFAP43 |
| ULBP2 |
| NPL |
| INHBE |
| FSD1L |
| TMEM120A |
| RASSF4 |
| FYTTD1 |
| CHCHD6 |
| DOT1L |
| KDM2B |
| PPP1R9B |
| NFKBID |
| ZFHX2 |
| ZC3H12C |
| DNAJC5B |
| RSPH1 |
| OTULIN |
| ESAM |
| FMNL3 |
| IZUMO4 |
| SFT2D1 |
| SDSL |
| LACTB |
| MIR155HG |
| LOC115110 |
| ALPK2 |
| RFFL |
| LRRC25 |
| FAM213B |
| DPCR1 |
| CFAP54 |
| NRG4 |
| TEKT5 |
| TRIM16L |
| DAB2IP |
| CXorf65 |
| GPR180 |
| LONRF2 |
| OLFML2A |
| MLKL |
| SAMD14 |
| SAMD9L |
| USP12 |
| PHACTR1 |
| LOC221946 |
| NEIL2 |
| LINC01619 |
| IL4I1 |
| LINC00515 |
| PRRT3 |
| LOC285628 |
| LOC285766 |
| HCG22 |
| TSPAN33 |
| SOWAHD |
| RASL11A |
| LINC00954 |
| PIM3 |
| ASTL |
| PIK3CD-AS1 |
| RAET1K |
| RPS16P5 |
| LOC730101 |
| TVP23A |
| LOC100130357 |
| LOC100130476 |
| LOC100130744 |
| LOC100506023 |
| MIR210HG |
| MIR4750 |
| ALMS1-IT1 |
| LOC100996583 |
| LINC01150 |
| MEF2C-AS1 |
| LOC101929709 |
| ABALON |

**Table S6: Common genes_UP**

| Gene | Ensembl ID |
| --- | --- |
| RXRA | ENSG00000186350 |
| CCL3 | ENSG00000277632 |
| CCL5 | ENSG00000271503 |
| SLAMF1 | ENSG00000117090 |
| STAT1 | ENSG00000115415 |
| EBI3 | ENSG00000105246 |
| IFI44L | ENSG00000137959 |
| CD274 | ENSG00000120217 |
| ACKR3 | ENSG00000144476 |
| GBP4 | ENSG00000162654 |

**Table S7:**

|  | Fwd | Probe | Rev |
| --- | --- | --- | --- |
| BamW | CCCAACACTCCACCACACC | CACACACTACACACACCCACCCGTCTC | TCTTAGGAGCTGTCCGAGGG |
| Pol | CTTTGGCGCGGATCCTC | CATCAAGAAGCTGCTGGCGGCC | AGTCCTTCTTGGCTAGTCTGTTGAC |

**Table S8: Tissue Cohort- EBV+ DLBCL**

| KEY | **Origin** | **Diagnosis** | **EBV** | **Gender** | **Age** | **COO** | **Treatment** | **Immune defects** | **Stage** | **Immunosuppressed** | **Site of biopsy** | **Location** |
| --- | --- | --- | --- | --- | --- | --- | --- | --- | --- | --- | --- | --- |
| S1 | Siena | DLBCL EBV+ | + | M | 64 | ABC |  |  |  |  | Nodal | Not specified |
| S2 | Siena | DLBCL EBV+ | + | F | 103 | ABC | metronomic therapy | No | II | No | Extranodal | Tonsil |
| S3 | Siena | DLBCL EBV+ | + | - | - | - | - | - | - | - | - | - |
| W1 | Wales | DLBCL EBV+ | + | M | 82 | ABC | RCVP | No | III | No | Nodal | Supraclavicular |
| W2 | Wales | DLBCL EBV+ | + | F | 62 | ABC | NA | No | II | No | Nodal | Supraclavicular |
| W3 | Wales | DLBCL EBV+ | + | F | 68 | ABC | RCHOP | No | III | Yes, methotrexate for rheumatoid arthritis | Nodal | Neck |
| W4 | Wales | DLBCL EBV+ | + | M | 85 | ABC | NA | No | III | No | Nodal | Groin |
| W5 | Wales | DLBCL EBV+ | + | F | 62 | ABC | NA | No | I | Yes, Myforti and steroids for SLE | Extranodal | Brain |
| W6 | Wales | DLBCL EBV+ | + | M | 79 | ABC | NA | No | IV | No | Nodal | Axilla |
| W7 | Wales | DLBCL EBV+ | + | F | 75 | ABC | Radiotherapy | Yes | II | No, CLL, hypogammaglobulinaemia | Extranodal | Tonsil |
| W8 | Wales | DLBCL EBV+ | + | - | - | - | - | - | - | - | - | - |
| W9 | Wales | DLBCL EBV+ | + | F | 83 | ABC | Radiotherapy | No | I | No | Extranodal | Calf |
| W10 | Wales | DLBCL EBV+ | + | M | 77 | ABC | NA | No | II | No | Extranodal | Adrenal gland |
| W11 | Wales | DLBCL EBV+ | + | F | 53 | ABC | RCHOP | No | II | Yes, multiple immunosuppressants for rheumatoid arthritis | Extranodal | Lung |
