# Supplementary material for: Loss of systemic anti-viral immunity and LMP1-driven suppressive myeloid tumour niches converge to shape the immunobiology of Epstein-Barr virus-positive diffuse large B-cell lymphoma
Source: Leukemia. 2026 Jun 10;40(8):1676–87. doi: 10.1038/s41375-026-02994-3 (PMC13421338; doi:10.1038/s41375-026-02994-3)
Supplement: Supplementary file 4 — Suppl Figure 4 [file 41375_2026_2994_MOESM4_ESM.pdf]

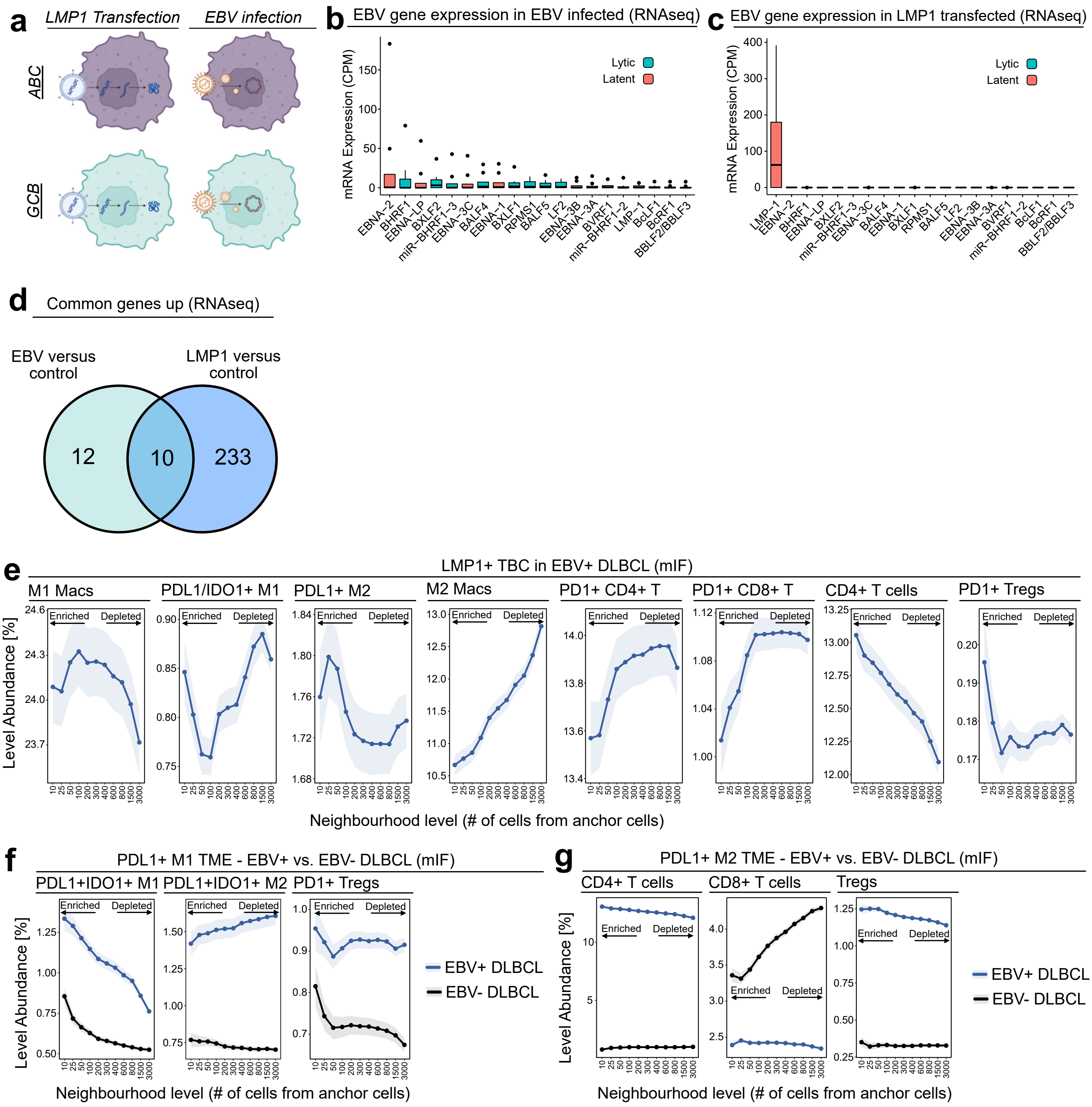
a
LMP1 Transfection
EBV infection
ABC
GCB
b
EBV gene expression in EBV infected (RNAseq)
mRNA Expression (CPM)
Lytic
Latent
EBNA-2
BHRF1
EBNA-LP
BXLF2
miR-BHRF1-3
EBNA-3C
BALF4
EBNA-1
BXLF1
RPMS1
BALF5
LF2
EBNA-3B
EBNA-3A
BVRF1
miR-BHRF1-2
LMP-1
BcLF1
BcRF1
BBLF2/BBLF3
c
EBV gene expression in LMP1 transfected (RNAseq)
mRNA Expression (CPM)
Lytic
Latent
LMP-1
EBNA-2
BHRF1
EBNA-LP
BXLF2
miR-BHRF1-3
EBNA-3C
BALF4
EBNA-1
BXLF1
RPMS1
BALF5
LF2
EBNA-3B
EBNA-3A
BVRF1
miR-BHRF1-2
BcLF1
BcRF1
BBLF2/BBLF3
d
Common genes up (RNAseq)
EBV versus control
LMP1 versus control
12
10
233
e
LMP1+ TBC in EBV+ DLBCL (mIF)
M1 Macs
PDL1/IDO1+ M1
PDL1+ M2
M2 Macs
PD1+ CD4+ T
PD1+ CD8+ T
CD4+ T cells
PD1+ Tregs
Enriched
Depleted
Level Abundance [%]
Neighbourhood level (# of cells from anchor cells)
f
PDL1+ M1 TME - EBV+ vs. EBV- DLBCL (mIF)
PDL1+IDO1+ M1
PDL1+IDO1+ M2
PD1+ Tregs
Level Abundance [%]
EBV+ DLBCL
EBV- DLBCL
Neighbourhood level (# of cells from anchor cells)
g
PDL1+ M2 TME - EBV+ vs. EBV- DLBCL (mIF)
CD4+ T cells
CD8+ T cells
Tregs
Level Abundance [%]
EBV+ DLBCL
EBV- DLBCL
Neighbourhood level (# of cells from anchor cells)
